# Copper Enhances Zinc-Induced Neurotoxicity and the Endoplasmic Reticulum Stress Response in a Neuronal Model of Vascular Dementia

**DOI:** 10.3389/fnins.2017.00058

**Published:** 2017-02-09

**Authors:** Ken-ichiro Tanaka, Masahiro Kawahara

**Affiliations:** Department of Bio Analytical Chemistry, Musashino UniversityNishitokyo-shi, Japan

**Keywords:** neurotoxicity, ER stress, ischemia, synapse, dementia, metal–metal interaction

## Abstract

Zinc (Zn), an essential trace element, is secreted by synaptic vesicles during neuronal excitation and plays several critical roles in neuronal information processing. However, excess Zn ion (Zn^2+^) is neurotoxic and has a causative role in the pathogenesis of vascular dementia. Here, we investigated the molecular mechanism of Zn^2+^-induced neurotoxicity by using immortalized hypothalamic neurons (GT1-7 cells), which are more vulnerable than other neuronal cells to Zn^2+^. We examined the effects of other metal ions on the Zn^2+^-induced neurotoxicity in these cells and found that sub-lethal concentrations of copper ion (Cu^2+^) markedly exacerbated Zn^2+^-induced neurotoxicity. The co-administration of Cu^2+^ and Zn^2+^ also significantly increased the expression of genes related to the endoplasmic reticulum's stress response, including *CHOP, GADD34*, and *ATF4*. Similar to Zn^2+^, Cu^2+^ is stored in presynaptic vesicles and secreted during neuronal excitation. Thus, based on our results, we hypothesize here that Cu^2+^ interacts with Zn^2+^ in the synapse to synergistically promote neuronal death and significantly influence the pathogenesis of vascular dementia.

## Introduction

Zinc (Zn) is essential for most organisms, and plays important roles in various physiological activities, including mitotic cell division, immune system functioning, protein, and DNA synthesis. Zn is also a co-factor for more than 300 enzymes or metalloproteins (Hambidge, 2000). Zn deficiency in human causes dwarfism, mental and physical development retardation, immune system dysfunction, learning disabilities, and taste and olfaction disorders (Prasad, 2009; Takeda and Tamano, 2012). Zn accumulates in the brain, especially in the hippocampus, amygdala, cerebral cortex, thalamus, and olfactory cortex (Frederickson et al., 2000). Although some Zn binds firmly to metalloproteins or enzymes, a substantial fraction (10% or more) either forms free Zn ions (Zn^2+^) or is loosely bound and is histochemically detectable by chelating agent staining. This chelatable Zn^2+^ is mainly stored in the presynaptic vesicles of specific excitatory glutamatergic neurons and is secreted into synaptic clefts along with glutamate during neuronal excitation (Frederickson et al., 1983).

Despite the necessity of Zn for normal neural function, excessive Zn^2+^ is toxic (Choi and Koh, 1998). In pathological conditions such as transient global ischemia or stroke, the interruption of blood flow and the resultant oxygen–glucose deprivation induces long-lasting membrane depolarization, causing the release of excessive Zn^2+^ and glutamate into synaptic clefts (Lee et al., 2000). Secreted Zn^2+^ is translocated into vulnerable neurons and causes apoptotic death of neurons and glial cells (Collins et al., 1989; Koh et al., 1996; Weiss et al., 2000; Calderone et al., 2004). These lines of evidence suggest that Zn^2+^ is a key mediator and modulator of delayed neuronal death after ischemia and that Zn^2+^ neurotoxicity is central to the pathogenesis of vascular dementia (VD), which is caused by a series of strokes or ischemic events (Shuttleworth and Weiss, 2011).

We have previously investigated molecular mechanism of Zn^2+^-induced neurotoxicity using immortalized hypothalamic neurons called GT1-7 cells. GT1-7 cells were developed by genetically targeting tumorigenesis to mouse hypothalamic neurons. These cells possess some neuronal characteristics, such as neurite extension as well as gonadotropin-releasing hormone secretion, and neuron-specific protein or receptors expressions (Mellon et al., 1990). We found that Zn^2+^ causes GT1-7 cell apoptotic death in a concentration and time-dependent manner and that GT1-7 cells are much more sensitive to Zn^2+^ than other neuronal cells (Koyama et al., 2011). We also previously demonstrated that Zn^2+^ induces a marked upregulation of endoplasmic reticulum (ER) stress-related genes, including CCAAT-enhancer-binding protein homologous protein (*CHOP)*, and growth-arrest- and DNA-damage-inducible gene 34 (*GADD34*), as well as calcium-related genes, including activity-regulated cytoskeleton-associated protein (*Arc*). We further determined that the ER stress pathway is involved in the molecular mechanism of Zn^2+^-induced neurotoxicity (Kawahara et al., 2013; Mizuno et al., 2015).

Considering that other trace elements, such as iron (Fe), copper (Cu), and manganese (Mn), are present and distributed to differing extents throughout the brain (Becker et al., 2010), it is possible that other metal ions interact in Zn^2+^-induced neurotoxicity. Indeed, we previously demonstrated that aluminum (Al^3+^) attenuates Zn^2+^-induced neurotoxicity by influencing the Zn^2+^-induced Ca^2+^ influx (Koyama et al., 2011). In the present study, we investigated the effect of other metal ions, including Cu^2+^, Mn^2+^, nickel (Ni^2+^), Fe^2+^, Fe^3+^, and Al^3+^, on Zn^2+^-induced neurotoxicity, and found that the coexistence of a sub-lethal concentration of Cu^2+^ significantly exacerbated Zn^2+^ neurotoxicity in GT1-7 cells.

## Materials and methods

### Reagents

Analytical grade pharmacological reagents were used. Metals including ZnCl_2_, NiCl_2_, and AlCl_3_ was purchased from Wako Pure Chemicals Ind. Ltd. (Tokyo, Japan). (Kyoto, Japan). Other metals including MnCl_2_, CuCl_2_, FeCl_2_, Fe(NO_3_)_3_, were purchased from Kanto Chemical Co., Ink. (Tokyo, Japan). Sodium dantrolene was purchased from Sigma Aldrich Co. LLC. (St. Louis, MO, USA). Antibody against actin (catalog number, SC-1616) and a donkey anti-goat horseradish peroxidase (HRP)-conjugated immunoglobulin G (IgG) secondary antibody (catalog number, SC-2056) were purchased from Santa Cruz Biotechnology (Santa Cruz, CA). The antibody against CHOP (catalog number, 5554S) was purchased from Cell Signaling Technology Japan (Tokyo, Japan). The mouse anti-rabbit IgG-HRP secondary antibody (Code: 211-032-171) was purchased from Jackson ImmunoResearch Laboratories, Inc. (West Grove, PA). RIPA buffer (20 mmol/L Tris-HCl [pH7.4], 200 mmol/L sodium chloride, 2.5 mmol/L magnesium chloride, 0.05% [w/v] NP-40 substitute) was purchased from WAKO Pure Chemicals (Tokyo, Japan).

### Cell culture

GT1-7 cells (provided by Dr. R. Weiner, University of California San Francisco, CA) were grown in Dulbecco's modified Eagle's medium/Ham's nutrient mixture F-12 (DMEM/F12) supplemented with 10% fetal bovine serum. After trypsin digestion, cells were resuspended in serum-free medium, distributed into culture dishes, and cultured in a humidified incubator (7% CO_2_) at 37°C (Kawahara et al., 2002). We used the same conditions throughout all experiments.

### Cell viability assay

Cell viability was assessed as previously described (Mizuno et al., 2015). Briefly, dissociated GT1-7 cells were distributed into 96-well culture plates at a concentration of 3 × 10^4^ cells per well in 200 μL of culture medium. After a 24 h incubation, the cells were treated with various compounds prior to the addition of ZnCl_2_ to the medium. After 24 h of exposure, cell viability was quantified using a WST-8 based cell counting kit (Dojindo, Kumamoto, Japan). The WST-8 assay used here is a modification of the 3-(4,5-dimethylthiazol-2-yl)-2,5-diphenyltetrazolium bromide (MTT) assay, which is widely used in the measurement of cell viability. The stable tetrazolium salt WST–8 is cleaved to a soluble formazan by cellular mitochondrial dehydrogenases in viable cells. Therefore, the amount of a formazan dye formed correlates to the number of viable cells (Ishiyama et al., 1996). Absorbance values of treated samples were measured against a blank control by using a Multiskan GO Spectrophotometer (Thermo Fisher Scientific Inc, Waltham, MA, USA) at 450 nm and 620 nm detection and reference wavelengths, respectively. The percentage absorbance of the samples relative to that of the control (no addition) was determined as the percentage viability of the cells. In each cell viability test, the effect of each concentration was examined at least in six wells (*n* = 6). The data are shown as mean percentages of cell viability compared with controls. Experiments were replicated at least two times.

### Real-time RT-PCR analysis

Zn-induced gene expression was assessed as previously described (Mizuno et al., 2015). Briefly, total RNA was extracted from GT1-7 cells grown in 6-well culture plates (7.5 × 10^5^ cells per well) using an RNeasy kit (Qiagen, Hilden, Germany) according to the manufacturer's protocol. Samples were reverse-transcribed (RT) using a PrimeScript® 1st strand cDNA Synthesis Kit (Takara Bio, Ohtsu, Japan). The synthesized cDNA was used in real-time RT-PCR experiments with SsoFast EvaGreen Supermix and analyzed with Bio-Rad's CFX96 real-time system and CFX Manager software (Hercules, CA). Specificity was confirmed by electrophoretic analysis of the reaction products and by the inclusion of template- or reverse transcriptase-free controls. To normalize the amount of total RNA present in each reaction, glyceraldehyde-3-phosphate dehydrogenase (*GAPDH*) cDNA was used as an internal standard. Primers were designed using the Primer-BLAST website by the National Center for Biotechnology Information. Primers sequences are described in Supplementary Table 1.

### Western blot assay

Zn-induced protein expression levels of CHOP and actin were assessed by western blot analysis. GT1-7 cells grown in 6-well culture plates (7.5 × 10^5^ cells per well) were lysed with RIPA buffer containing both protease and phosphatase inhibitors (catalog number, 87786 and 78420, Thermo Fisher Scientific Inc.). Protein concentrations were measured using the Bradford Reagent (Bio-Rad, Hercules, CA, USA). The samples were applied to NuPAGE Novex 4%–12% Bis-Tris protein gels (Thermo Fisher Scientific Inc.), and electrophoresed at a constant voltage of 180 V. The proteins were transferred to polyvinylidene difluoride membranes using an iBlot 7-Min Blotting System (Thermo Fisher Scientific Inc.). Membranes were blocked with 5% non-fat dry milk at room temperature for 1 h, incubated with rabbit anti-CHOP antibody (1:1,000 dilution) or goat anti-actin antibody (1:1,000) in 5% bovine serum albumin, 1 × TBS and 0.1% Tween 20 overnight, and then finally incubated with mouse anti-rabbit IgG-HRP secondary antibody (1:10,000) or donkey anti-goat IgG-HRP secondary antibody (1:10,000) in 1 × TBS and 0.1% Tween 20 for 1 h. The resultant bands were visualized using SuperSignal West Dura Extended Duration Substrate (Thermo Fisher Scientific Inc.), and band intensities were quantitated using ImageJ software (version 1.39u, National Institutes of Health, Bethesda, MD, USA). The band intensity of each protein was normalized with respect to that of actin.

### Statistical analysis

All values are expressed as the mean ± standard deviation (*SD*). Homoscedasticity of data were verified by Levene test, then, data were examined using one-way analysis of variance followed by Tukey's test or Games-Howel test for unpaired results (including controls). All statistical analyses were conducted using SPSS Statistics 24 software. Differences were considered to be significant for values of *P* ≤ 0.05.

## Results

### Effects of various metals on Zn^2+^-induced neurotoxicity

**Figure 1:**
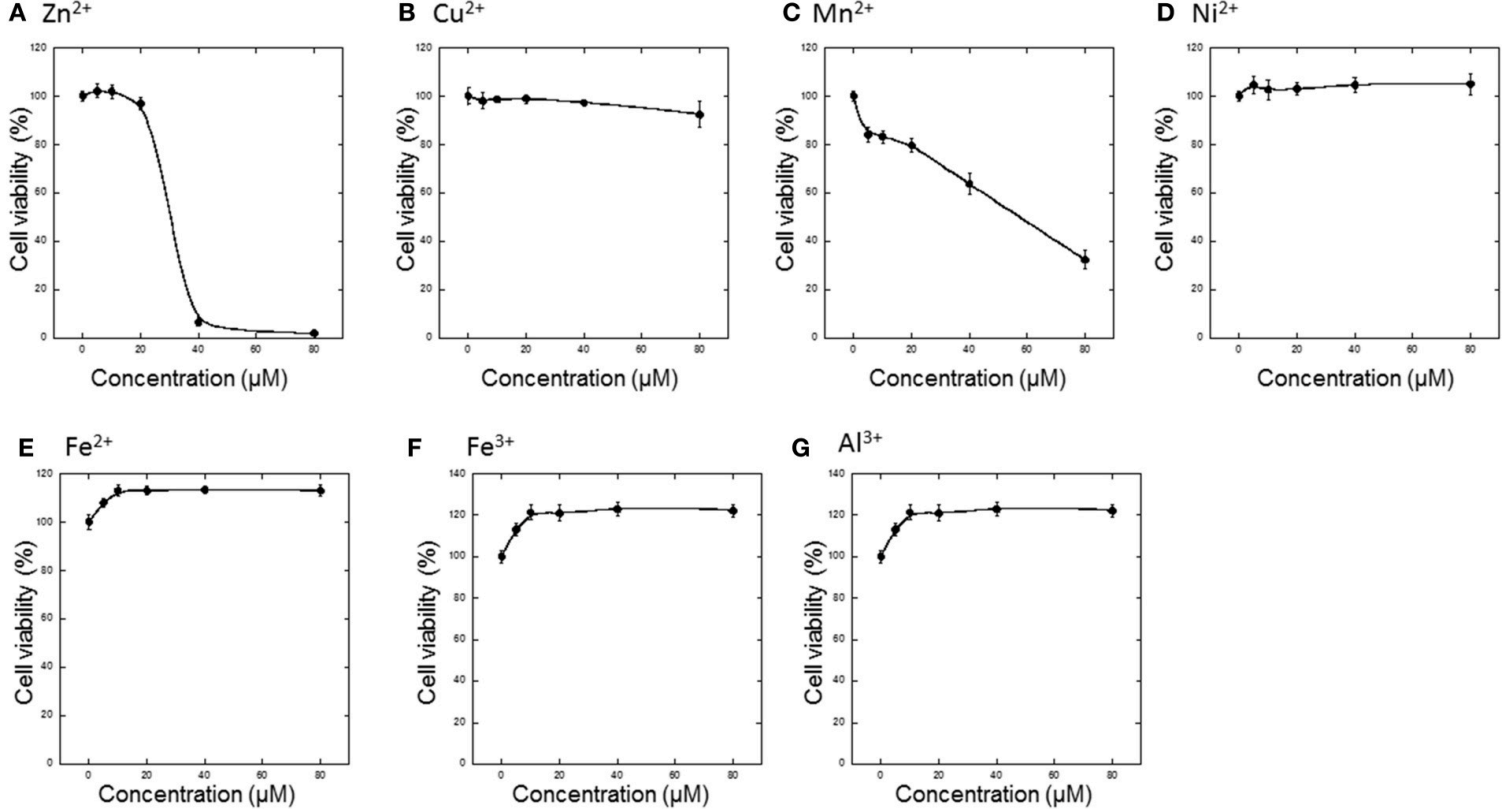
**Effects of various metals on the neurotoxicity of GT1-7 cells. (A)** ZnCl_2_, **(B)** CuCl_2_, **(C)** MnCl_2_, **(D)** NiCl_2_, **(E)** FeCl_2_, **(F)** Fe(NO_3_)_3_, or **(G)** AlCl_3_ was administered to GT1-7 cells. After 24 h, cell viability was determined using the WST-8 method. Six wells were exposed to the same experimental conditions (*n* = 6). Data are presented as means ± *SD* of cell viability. Experiments were replicated at least two times.

First, various metals, including divalent (Zn^2+^, Cu^2+^, Mn^2+^, Ni^2+^, Fe^2+^) and trivalent (Fe^3+^ and Al^3+^) ions, were applied to GT1-7 cells, and cell viability was determined 24 h later (Figures 1A–G). The addition of 5−80 μM Ni^2+^, Fe^2+^, Fe^3+^, or Al^3+^ did not cause cell death. However, Zn^2+^ significantly decreased the viability of GT1-7 cells in a concentration-dependent manner. The viability of cells exposed to 40 μM of Zn^2+^ was 6.7 ± 1.8% (mean ± *SD, n* = 6) of control, which was no addition of metals to cells. We estimated the LD_50_ of Zn^2+^ to be ~35 μM. Application of Mn^2+^ and Cu^2+^ also decreased cell viability, although the toxicity induced by these metals was less than that observed with Zn^2+^ (Figures 1B,C).

**Figure 2:**
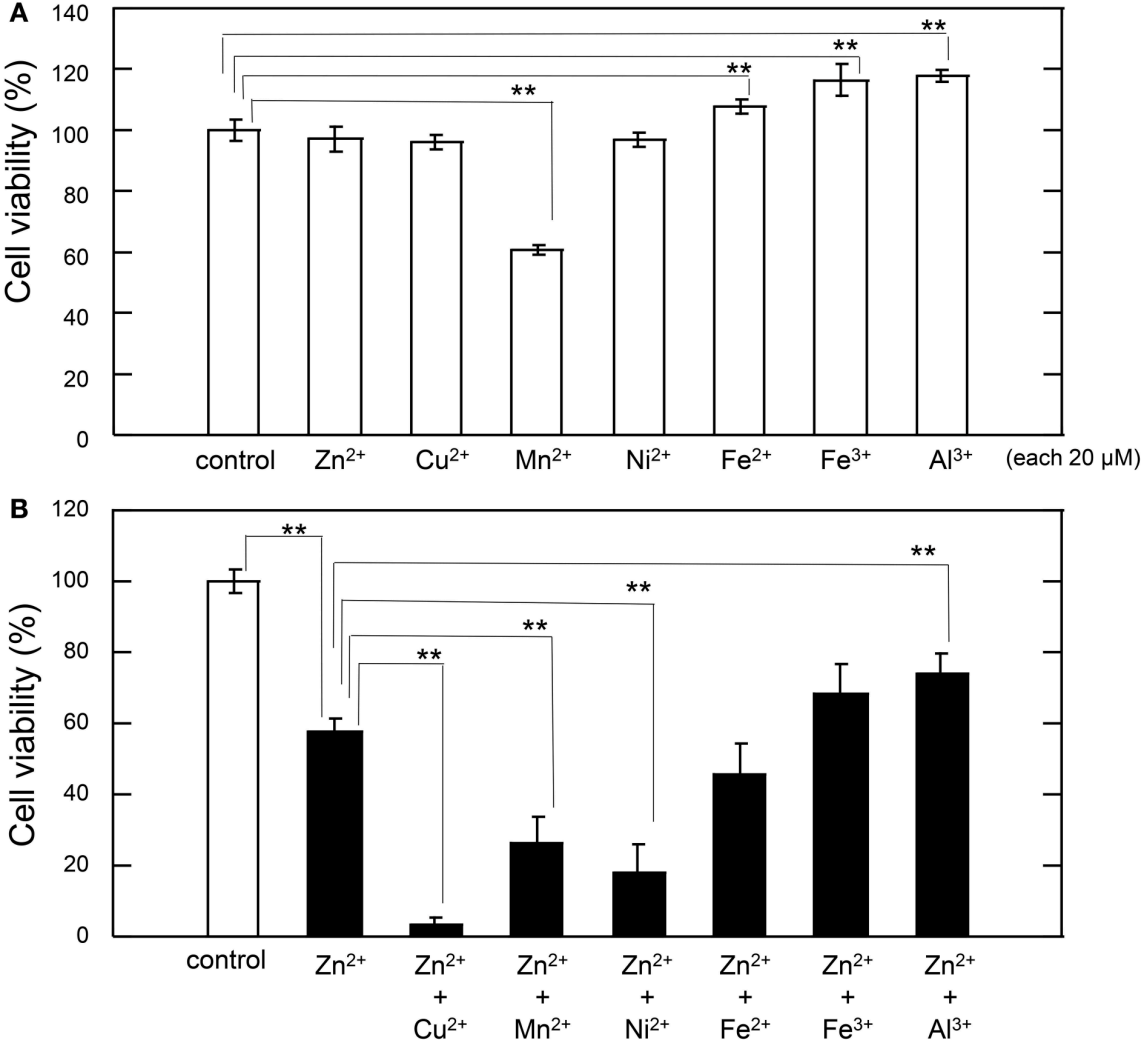
**Effects of various metals on Zn^**2+**^-induced neurotoxicity. (A)** GT1-7 cells were exposed to 20 μM ZnCl_2_, CuCl_2_, MnCl_2_, NiCl_2_, FeCl_2_, Fe(NO_3_)_3_, or AlCl_3_. After 24 h, cell viability was determined using the WST-8 method. Six wells were exposed to the same experimental conditions (*n* = 6). Data are presented as means ± *SD* of cell viability. Experiments were replicated at least two times. ^*^p < 0.05, ^**^p < 0.01. **(B)** GT1-7 cells were exposed to 20 μM CuCl_2_, MnCl_2_, NiCl_2_, FeCl_2_, Fe(NO_3_)_3_, or AlCl_3_ with 30 μM ZnCl_2_ in the same experimental condition in **(A)**. After 24 h, cell viability was determined using the WST-8 method. Six wells were exposed to the same experimental conditions (*n* = 6). Data are presented as means ± *SD* of cell viability. Experiments were replicated at least two times. ^*^p < 0.05, ^**^p < 0.01.

Thus, we tested the interaction between sub-lethal concentrations of these metals and Zn. The cell viability after exposure of GT1-7 cells to each metal ion alone (20 μM) is shown in Figure 2A. The exposure to Mn^2+^ was slightly toxic, with a cell viability of 60.6 ± 1.7%. Under serum-free conditions, the addition of Fe^3+^, Fe^2+^, or Al^3+^ increased in cell viability. We next exposed the cells to each metal ion in the presence of 30 μM Zn^2+^ (Figure 2B). After 24 h of exposure to 30 μM Zn^2+^, cell viability was decreased to 57.5% ± 3.9%. The addition of 20 μM Al^3+^ significantly improved cell viability (74.0 ± 5.6%). By contrast, the addition of 20 μM of Fe^2+^ or Fe^3+^ resulted in no significant change. The simultaneous administration of the divalent ions Cu^2+^, Mn^2+^, or Ni^2+^ with Zn^2+^ caused a synergistic effect, inducing greater neurotoxicity than that observed by the addition of either metal alone. Of the four divalent ions examined, the synergistic effect of Zn^2+^ and Cu^2+^ was most marked with cell viability decreasing to 3.2 ± 2.7% after co-administration of 20 μM Cu^2+^ and 30 μM Zn^2+^, compared with 57.5 ± 3.9% for Zn^2+^ alone. Cell viability decreased after co-administration of Ni^2+^ and Zn^2+^ to 18.0 ± 8.0%, and after co-administration of Mn^2+^ and Zn^2+^ to 26.0 ± 7.4% (compared with 60.6 ± 1.7% for Mn^2+^ alone). These results exhibited that sub-lethal concentrations of Cu^2+^, Mn^2+^, and Ni^2+^ with Zn^2+^ caused the synergistic effects in Zn^2+^-induced neurotoxicity and that the effects of Cu^2+^ is most significant compared with other ions.

### Cu^2+^-enhanced Zn^2+^ neurotoxicity

**Figure 3:**
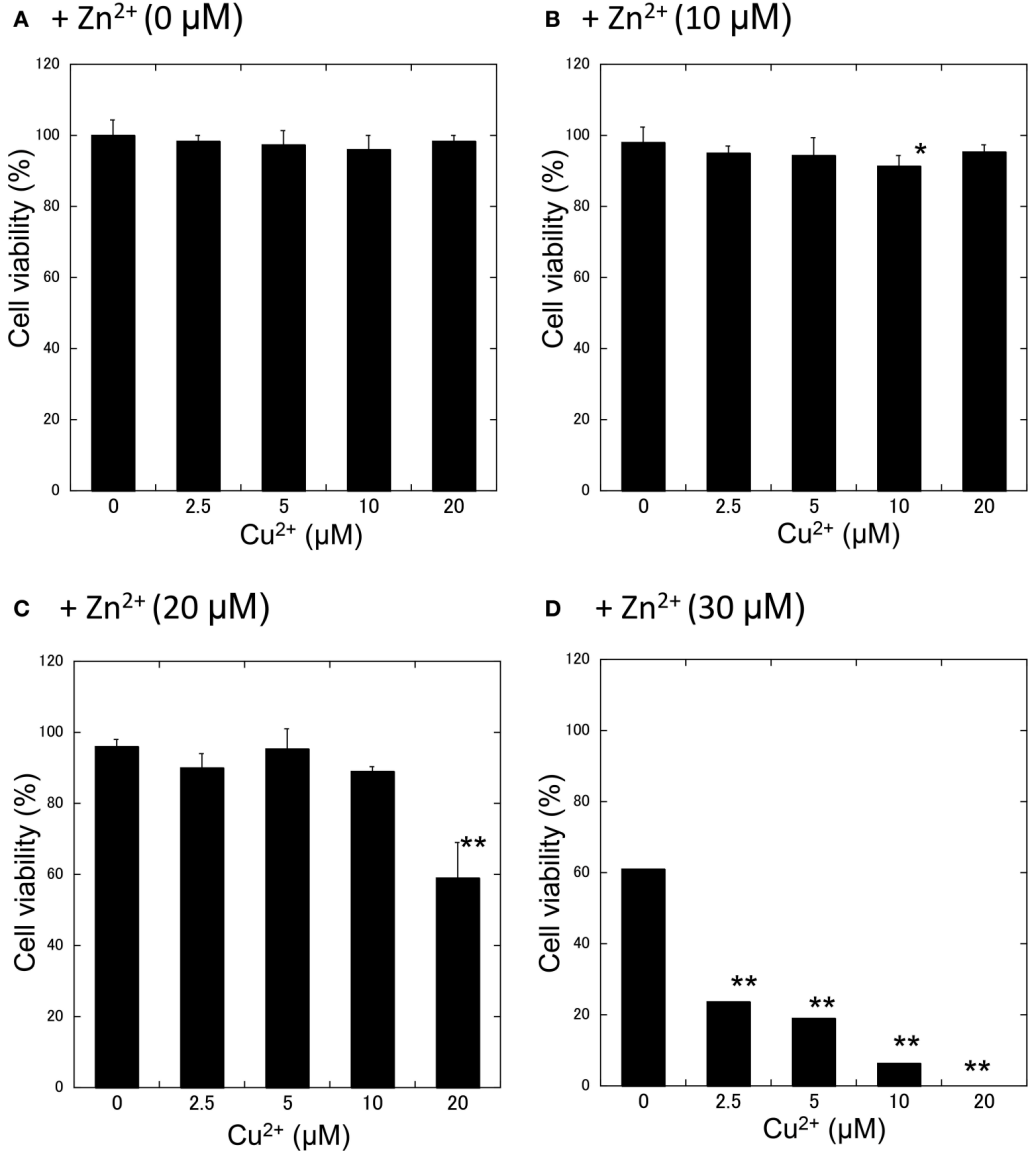
**Effects of Cu^**2+**^ on Zn^**2+**^-induced neurotoxicity**. Various concentrations of CuCl_2_ (0~20 μM) without ZnCl_2_ **(A)** or with 10 μM **(B)**, 20 μM **(C)**, or 30 μM ZnCl_2_ **(D)** were administered to GT1-7 cells. After 24 h, cell viability was determined using the WST-8 method. Six wells were exposed to the same experimental conditions (*n* = 6). Data are presented as means ± *SD*. Experiments were replicated at least two times. ^*^p < 0.05, ^**^p < 0.01 vs. CuCl_2_ (0 μM).

We further investigated the synergistic effects of Cu^2+^ and Zn^2+^ on neurodegeneration. First, GT1-7 cells were exposed to various concentrations (5−20 μM) of Cu^2+^ with increasing concentrations of Zn^2+^ (0−30 μM: Figure 3). The addition of 2.5 μM Cu^2+^ with 30 μM Zn^2+^ (Cu^2+^:Zn^2+^ molar ratio, 1:12) significantly decreased cell viability compared with that of Zn^2+^ alone. The co-administration of 5 μM Cu^2+^ with 30 μM Zn^2+^ (Cu^2+^:Zn^2+^ molar ratio, 1:6) decreased cell viability to 19.1 ± 4.7%. The addition of 20 μM Cu^2+^ with 20 μM Zn^2+^ (Cu^2+^:Zn^2+^ molar ratio, 1:1) decreased cell viability to 58.8 ± 9.9%, which was significantly lower than that of Zn^2+^ alone (96.1 ± 9.9%).

### Gene expressions is altered after Cu^2+^ and Zn^2+^ co-administration

**Figure 4:**
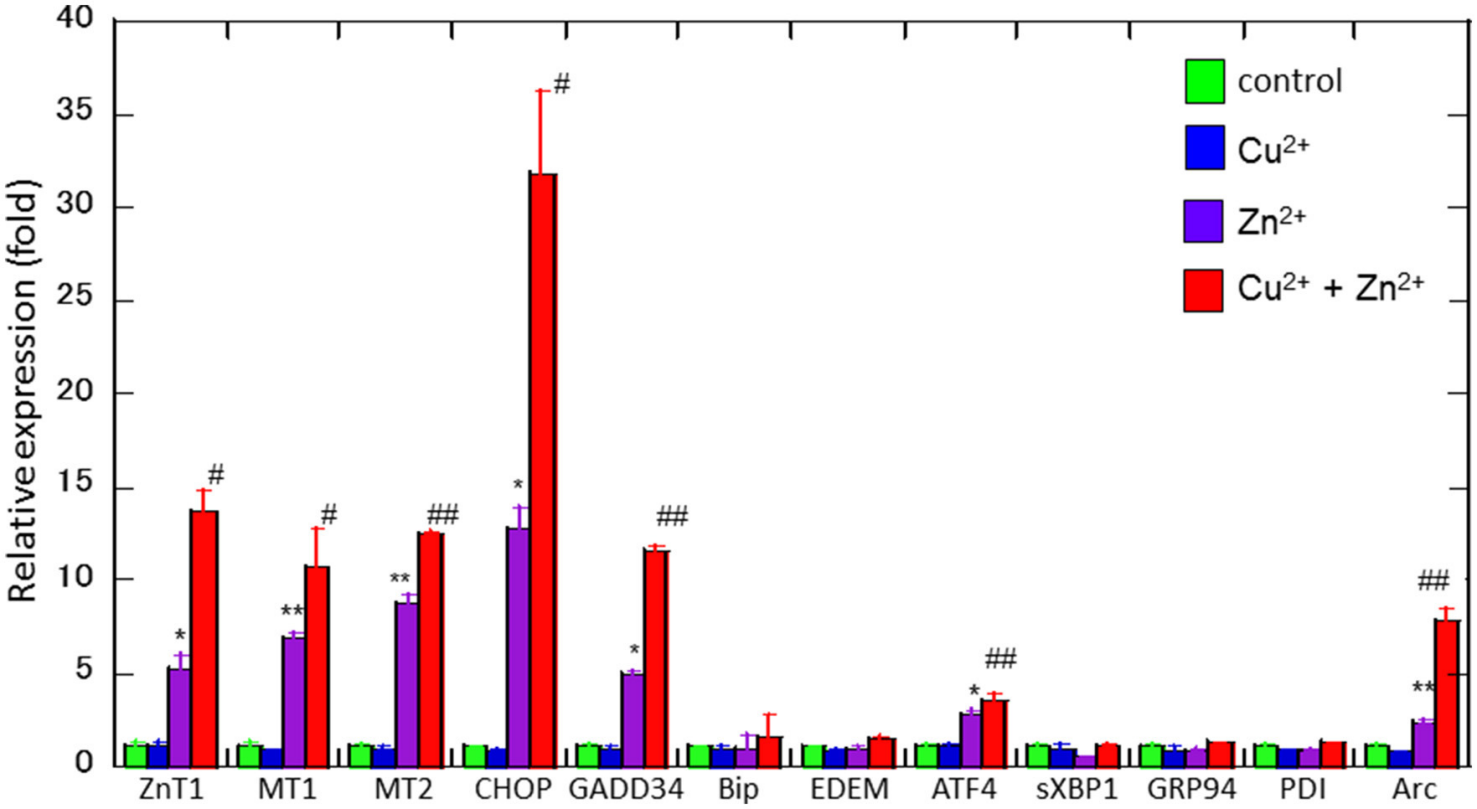
**Effects of Cu^**2+**^ on Zn^**2+**^-induced gene expression**. Expression levels of *CHOP, GADD34, Arc, Bip, ATF4, EDEM, sXBP1, GRP94, PDI, ZnT-1, MT1*, and *MT2* were analyzed using real-time RT–PCR. Gene expression levels were normalized with *GAPDH*. Data are presented as means ± *SD* (*n* = 3). Experiments were replicated at least two times. ^*^p < 0.05, ^**^p < 0.01 compared with control; ^#^*p* < 0.05, ^##^*p* < 0.01 compared with Zn^2+^.

**Figure 5:**
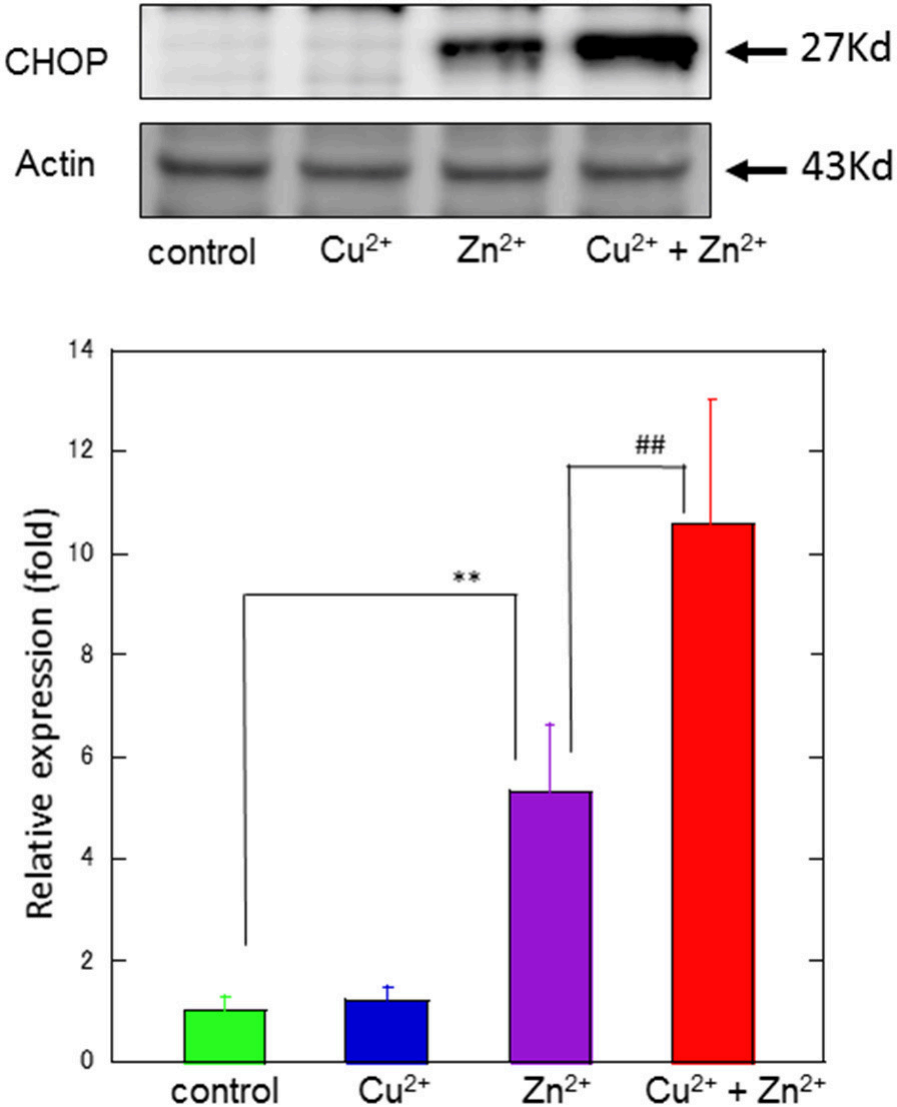
**Effects of Cu^**2+**^ on Zn^**2+**^–induced CHOP protein expression**. The expression of CHOP protein in GT1-7 cells was assayed using western blotting. GT1-7 cells were treated with 30 μM Zn^2+^, 20 μM Cu^2+^, or a mixture of 30 μM Zn^2+^ with 20 μM Cu^2+^ for 4 h. The blot was probed with an anti-CHOP antibody, and the band intensities were analyzed using ImageJ software. The values shown were obtained by dividing the intensity of the respective band by that of the standard band. Data are presented as means ± *SD* (*n* = 3). ^**^p < 0.01 compared with control; ^##^*p* < 0.01 compared with Zn^2+^. Experiments were replicated at least two times.

To study the molecular mechanism for the Cu^2+^-enhanced Zn^2+^ neurotoxicity, we analyzed gene expression in these cells using real-time RT-PCR (Figure 4). For this purpose, based on our previous results and possible apoptotic pathways (Mizuno et al., 2015), we selected several metal-related genes, including zinc transporter 1 *(ZnT-1)*, metallothionein 1*(MT1)*, and metallothionein 2 (*MT2)*, Ca^2+^-related (*Arc*) gene, and ER stress-related genes [*CHOP, GADD34*, activating transcription factor 4 (*ATF4*), immunoglobulin binding protein (*Bip*), ER degradation-enhancing α-mannosidase-like protein (*EDEM*), spliced X-box binding protein-1 (*sXBP1*), glucose-regulated protein 94 (*GRP94*), and protein disulfide isomerase (*PDI*)]. After 4 h of exposure to 30 μM Zn^2+^ alone, the expression of metal-related genes, including *ZnT-1, MT1*, and *MT2*, increased. In addition, enhanced expression levels for *Arc, CHOP, GADD34*, and *ATF4 genes*, were observed. By contrast, other ER stress-related genes including *Bip, EDEM, sXBP1, GRP94*, and *PDI* did not exhibit significant changes. The exposure of cells to 20 μM Cu^2+^ alone did not induce significant changes in these genes. However, a synergistic increase in gene expression levels of *Arc, CHOP*, and *GADD34* were observed in cells co-exposed to Cu^2+^ and Zn^2+^. In particular, the relative expression of *CHOP* after co-administration of Cu^2+^ and Zn^2+^ was 32.0 ± 4.6-fold (mean ± *SD, n* = 3), which was significantly increased compared with that of Zn^2+^ alone (12.5 ± 1.1-fold). We also used western blotting analysis to determine the amount of CHOP protein, which is responsible for initiating an apoptotic cascade. We found that the amount of CHOP protein was significantly increased after co-administration of Cu^2+^ and Zn^2+^, compared to Zn^2+^ alone. The amount of CHOP protein after the co-administration of Cu^2+^ and Zn^2+^ was 10.6 ± 2.5-fold, compared with that following administration of Zn^2+^ alone, 5.3 ± 1.3-fold) (Figure 5). These results indicate that expression of *CHOP, GADD34*, and *ATF4* genes were enhanced by co-administration of Cu^2+^ and Zn^2+^, and other ER stress-related genes were not. Although the administration of Zn^2+^ alone upregulates these ER stress-related genes, Cu^2+^ alone did not. Several factors involved in the ER stress pathway *(Bip, EDEM, sXBP1, GRP94*, and *PDI*) were not upregulated after exposure to Zn^2+^ alone as well as to Cu^2+^ and Zn^2+^ together. Our previous results exhibit that the expression of metal-related genes, such as *ZnT-1, MT1, MT2*, as well as the expression of ER stress-genes, including *GADD34* and *CHOP*, were upregulated after exposure to Zn^2+^ (Kawahara et al., 2013). We have also demonstrated that substances which attenuate Zn^2+^-induced neurotoxicity, such as carnosine or histidine, decreased the expression of these ER stress related genes (Mizuno et al., 2015). Furthermore, dantrolene, an inhibitor of ER stress, attenuated Zn^2+^-induced neurotoxicity (Supplementary Figure 1). Based on these findings, it is possible that Cu^2+^ potentiate Zn^2+^-induced ER stress pathways, and thereafter enhance Zn^2+^-induced neurotoxicity.

## Discussion

Our present results demonstrated that sub-lethal concentrations of Cu^2+^ markedly enhanced the Zn^2+^-induced neurotoxicity of GT1-7 cells. Zn is the second most abundant trace element in the brain. It is accumulated in presynaptic vesicles and released during neuronal excitation. The secreted Zn^2+^ plays crucial roles in information processing, synaptic plasticity, learning, and memory (Ueno et al., 2002; Takeda et al., 2014). Although the amount of free Zn^2+^ secreted from synaptic vesicles is controversial (Frederickson et al., 2006), several studies have estimated the concentration of Zn^2+^ in the synaptic cleft to be 1–100 μM (Sensi et al., 1997; Vogt et al., 2000; Kay, 2006; Zhang et al., 2012).

The third most abundant trace elements in the brain is Cu. Recent studies suggest that intracellular Cu^2+^ accumulates in synaptic vesicles and is then released into the synaptic cleft during neuronal excitation, similar to Zn^2+^ (Opazo et al., 2014; D'Ambrosi and Rossi, 2015). The concentration of Cu^2+^ in the synaptic cleft is estimated to be approximately 2−15 μM (Hopt et al., 2003). The translocated Cu^2+^ influences various receptors, including the NMDA-type glutamate receptor, AMPA-type glutamate receptor, and GABA receptor, and contributes to the modulation of neuronal excitability, similar to Zn^2+^ (Mathie et al., 2006; Gaier et al., 2013; Dodani et al., 2014). Because the concentrations used in our experiments were similar to those observed in synaptic clefts, it is likely that synergistic actions of Zn^2+^ and Cu^2+^ occur in the neurodegenerative processes of VD.

Our data also suggested that other divalent cations, including Mn^2+^ and Ni^2+^ exhibited synergistic effects on Zn^2+^-induced neurotoxicity. However, their effects were markedly lower than those induced by Cu^2+^, as shown in Figure 2. Mn^2+^ also exists in the brain and is essential for of neurotransmitter synthesis and as a component of superoxide dismutase (Aschner, 2000). However, excess Mn^2+^ is neurotoxic and causes Parkinson disease-like syndrome (Kwakye et al., 2015). We used Ni^2+^ as a model of a non-essential divalent cation. Ni^2+^ is reportedly toxic, as it inhibits Ca^2+^ homeostasis and Ca^2+^-mediated cell signaling (Guo et al., 2015; Saito et al., 2016). Additionally, Fe is the most abundant trace element in the brain. However, the concentration of free Fe ions, as free Fe ions are toxic (Muñoz and Humeres, 2012; Núñez et al., 2012). Thus, it is unlikely that Zn^2+^ interacts with these metals, given their low physiological concentrations in the brain.

We have previously investigated the molecular mechanisms of Zn^2+^-induced neurotoxicity using GT1-7 cells as a neuronal model for VD (Kawahara et al., 2002). Many researchers have investigated Zn^2+^ neurotoxicity *in vitro*, mainly using primary cultured neurons from rat cerebral cortex or hippocampus, or PC-12 cells, a pheochromocytoma cell line (Kim et al., 2000; Sheline et al., 2000). However, both glutamate and Zn^2+^ are neurotoxic, distinguishing the effects of Zn^2+^ and glutamate by using neuronal cells that possess glutamate receptors has proved difficult. GT1-7 cells either lack or possess low levels of ionotropic glutamate receptors and do not exhibit glutamate toxicity (Mahesh et al., 1999; Loikkanen et al., 2003). Furthermore, we found that GT1-7 cells are much more sensitive to Zn^2+^ than other neuronal cells, including PC-12 cells, B-50 cells (a neuroblastoma cell line), or primary cultured neurons of the rat cerebral cortex or hippocampus (Koyama et al., 2011). These properties make the GT1-7 cell line an excellent model system for investigating Zn^2+^-induced neurotoxicity and VD.

The results of our previous studies have suggested that several substances, including sodium pyruvate, sodium citrate, Al^3+^, carnosine (β-alanyl-L-histidine), and histidine, attenuate Zn^2+^-induced neurotoxicity of GT1-7 cells (Kawahara et al., 2002, 2007, 2013). Based on such evidence, we hypothesize that Zn^2+^-induced ER stress plays a central role in neurodegenerative processes. ER stress is critically involved in various neurological disorders, such as cerebral ischemia, Alzheimer's disease, and prion diseases (Xin et al., 2014; Torres et al., 2015; Zhang et al., 2015).

Our present results obtained from real-time RT-PCR analyses demonstrated that co-administration of Zn^2+^ and Cu^2+^ caused markedly increased the expression of the ER stress-related genes *CHOP, GADD34*, and *ATF4* compared to control. Thus, it is highly likely that low concentrations of Cu^2+^ potentiate Zn^2+^-induced ER stress pathways, and thereafter promote Zn^2+^-induced neurotoxicity. ER stress triggers the unfolded protein response, which is distinguished by three signaling proteins (ER stress sensors) termed inositol-requiring enzyme-1α (IRE1α), protein kinase R (PKR)-like ER kinase (PERK), and activating transcription factor-6 (ATF6) (Sano and Reed, 2013). Upon activation, IRE1α, PERK, and ATF6 induce various signal transduction events (Rozpedek et al., 2016). The phosphorylation of the α subunit of eukaryotic translation initiation factor (elF2α) is mediated by PERK and then influences the translation of ATF4. ATF4 is a transcription factor that drives the expression of CHOP and GADD34. Based on our present results, it is plausible that the *PERK*-related pathway may be involved in Zn^2+^-induced ER stress. CHOP directly induces apoptosis or mediates the activation of GADD34 (Li et al., 2014; Iurlaro and Mu-oz-Pinedo, 2016). GADD34 is upregulated after ischemia and reportedly causes neurotoxicity after traumatic brain injury (McCabe et al., 2007). Further research will be needed to elucidate the involvement of other pathways, including mitochondrial dysfunctions and the Ca^2+^ dyshomeostasis in the synergistic actions of Cu^2+^ and Zn^2+^.

**Figure 6:**
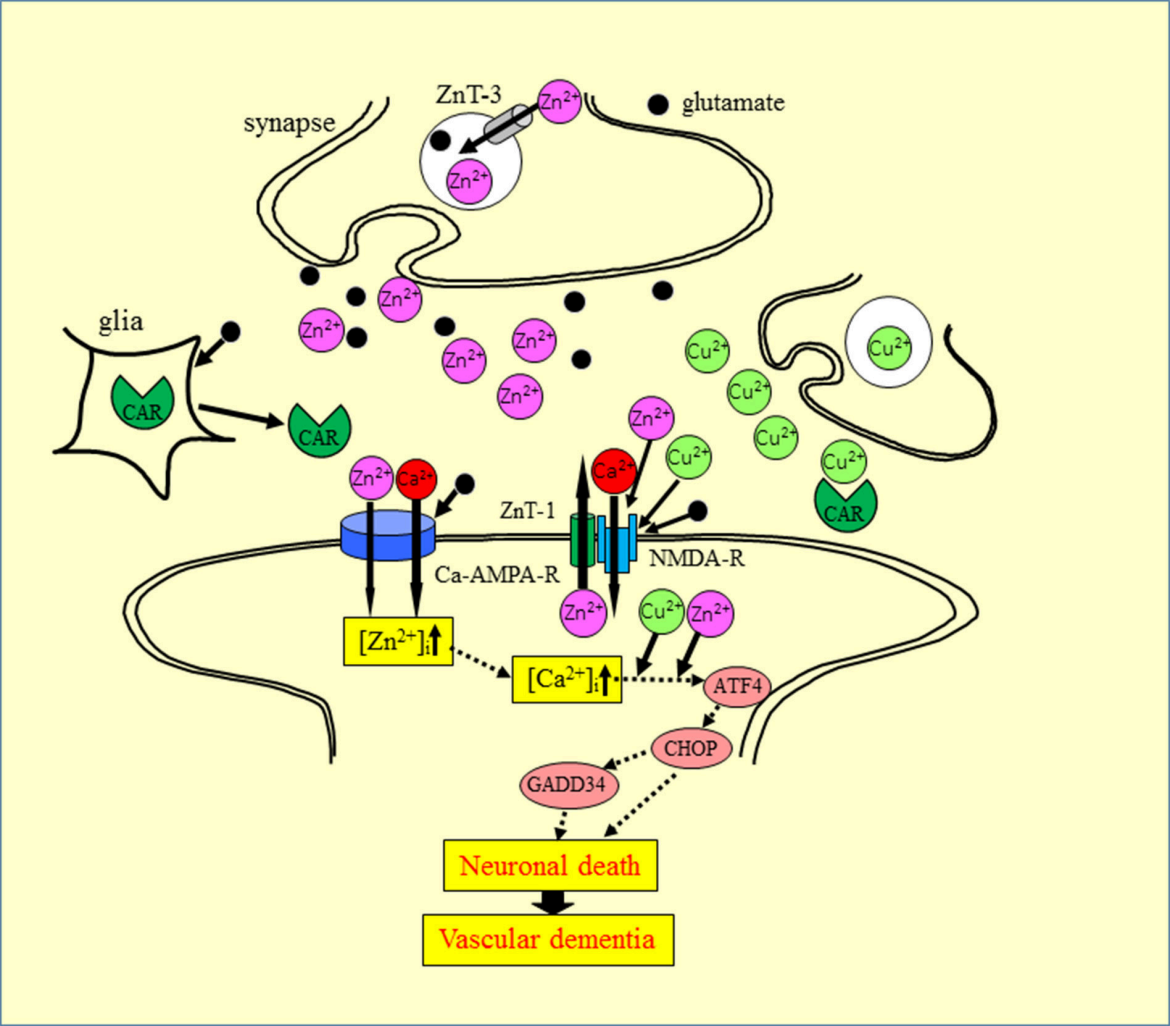
**Proposed mechanism for the synergistic interaction between Zn^**2+**^ and Cu^**2+**^ at the synapse**. Zn and glutamate accumulate in synaptic vesicles and are released into synaptic clefts during neuronal excitation. Zn^2+^ regulates Ca^2+^ influx through NMDA-type glutamate receptors, modulates neuronal information, and is implicated in the maintenance of synaptic plasticity and memory formation, similar to Ca^2+^. Zn^2+^ enters target neurons via voltage-dependent Ca^2+^ channels, NMDA-type glutamate channels, and Ca^2+^-permeable AMPA/kainate channels. The increased intracellular Zn^2+^ induces ER stress pathways and triggers apoptotic pathways. The ZnT-1 Zn transporter regulates Zn homeostasis and is localized to post-synaptic membranes that express NMDA-type glutamate receptors. Carnosine is released from glial cells into synaptic clefts, and is thought to regulate excess Zn. ZnT-1, zinc transporter 1; ZnT-3, zinc transporter 3; AMPA-R, AMPA-type glutamate receptor; NMDA-R, NMDA-type glutamate receptor; CAR, carnosine.

Based on our findings and the aforementioned evidence, we propose a hypothesis offering a potential mechanism for the interaction of Zn^2+^ and Cu^2+^ at the synapse (Figure 6). During aberrant conditions, such as those occurring during transient global ischemia, excess Zn^2+^ secreted into the synaptic cleft, is translocated into target neurons *via* voltage-dependent Ca^2+^ channels, NMDA-type glutamate channels, and Ca^2+^-permeable AMPA/kainate channels (Jia et al., 2002). The excess Zn^2+^ triggers several apoptotic pathways, including that involving ER stress. Considering that the synaptic cleft can be conceptualized as a small cylinder with a height of 20 nm and a radius of 200 nm (Schikorski and Stevens, 1997), it is highly likely that the Cu^2+^ that is released in the synaptic clefts, as well as Zn^2+^, spills into the neighboring Zn^2+^-containing synapses. The excess Cu^2+^ could then enhance Zn^2+^-induced neurotoxicity. This plausible mechanism may explain the discrepant vulnerability to ischemia observed across different neuronal regions (Collins et al., 1989).

This hypothesis suggests that the synapse may be the primary target affected by Zn-plus Cu-induced neurotoxicity and in the pathogenesis of VD. Thus, factors regulating metal homeostasis may be important. Recent studies have suggested that ZnT-1 is located in post-synaptic membranes (Sindreu et al., 2014), and plays important roles in the efflux of intracellular Zn^2+^ and binds with NMDA-type glutamate receptors to regulate their functions (Mellone et al., 2015). Carnosine is another factor that controls metal homeostasis in the synapse. Carnosine attenuates Zn^2+^-induced neurotoxicity in GT1-7 cells and cultured rat hippocampal neurons. Carnosine is a naturally occurring dipeptide and provides various benefits, such as pH balance, as well as anti-glycation, antioxidant, anti cross-linking, and anti-fatigue activities (Boldyrev et al., 2013). Carnosine is synthesized in glial cells and secreted into the synaptic cleft (De Marchis et al., 2000). Carnosine attenuates ischemia-induced neuronal death in experimental animals (Pekcetin et al., 2009). Based on this evidence for the roles of carnosine, we previously published a patent for carnosine as a protective drug in VD (Kawahara et al., 2007).

## Conclusion

Our proposed model provides a plausible molecular mechanism elucidating ischemia-induced neurotoxicity and the pathogenesis of VD and may contribute to developing novel treatments in VD. Further research exploring the molecular mechanisms underlying Cu^2+^ and Zn^2+^ synergistic neurotoxicity is warranted and may lead to novel therapeutic approaches in neurodegenerative diseases treatments.

## Author contributions

Participated in research design: KT and MK; Conducted experiments: KT; Contributed new reagents or analytic tools: KT; Performed data analysis: KT; Wrote or contributed to the writing of the manuscript: KT and MK.

## Funding

This work was partially supported by a Grant-in Aid for Scientific Research from the Ministry of Education, Culture, Sports, Science, and Technology of Japan. (JSPS Kakennhi Grant No. 26460177).

### Conflict of interest statement

The authors declare that the research was conducted in the absence of any commercial or financial relationships that could be construed as a potential conflict of interest.

## Supplementary material

Supplementary Figure 1 **Effects of dantrolene on Zn^**2+**^-induced neurotoxicity GT1-7 cells were exposed to 30 μM ZnCl_**2**_ without or with 5~10 μM dantrolene**. After 24 h, cell viability was determined using the WST-8 method. Six wells were exposed to the same experimental conditions (*n* = 6). Data are presented as means ± *SD* of cell viability. Experiments were replicated at least two times. ^**^*p* < 0.01 compared with control; ^##^*p* < 0.01 compared with Zn^2+^.

Supplementary Table 1 **Sequences of primers**.
