# Supplementary figures and images for: Copper Enhances Zinc-Induced Neurotoxicity and the Endoplasmic Reticulum Stress Response in a Neuronal Model of Vascular Dementia
Source: Front Neurosci. 2017 Feb 9;11:58. doi: 10.3389/fnins.2017.00058 (PMC5299027; doi:10.3389/fnins.2017.00058)

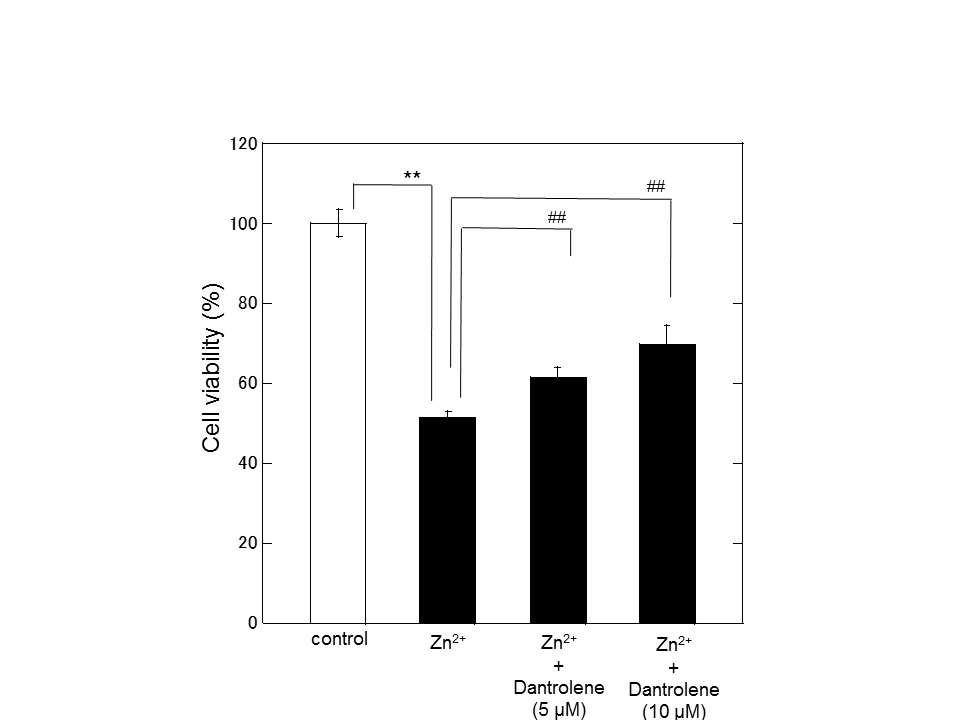

Supplement: Supplementary Figure 1 — Effects of dantrolene on Zn2+-induced neurotoxicity GT1-7 cells were exposed to 30 μM ZnCl2 without or with 5~10 μM dantrolene. After 24 h, cell viability was determined using the WST-8 method. Six wells were exposed to the same experimental conditions (n = 6). Data are presented as means ± SD of cell viability. Experiments were replicated at least two times. **p < 0.01 compared with control; ##p < 0.01 compared with Zn2+. [file Image1.jpg]
