# Supplementary material for: Copper Enhances Zinc-Induced Neurotoxicity and the Endoplasmic Reticulum Stress Response in a Neuronal Model of Vascular Dementia
Source: Front Neurosci. 2017 Feb 9;11:58. doi: 10.3389/fnins.2017.00058 (PMC5299027; doi:10.3389/fnins.2017.00058)
Supplement: Supplementary Table 1 — Sequences of primers. [file Table1.PDF]

Name	*Forward*	*Reverse*
GAPDH	AACTTTGGCATTGTGGAAGG	ACACATTGGGGGTAGGAACA
ZnT1	TGTTCATGGTGCTGGAGGTG	ATCCAGCCGAACGTGTTCTT
MT1	CCAACTGCTCCTGCTCCAC	AGCAGCAGCTCTTCTTGCAG
MT2	CGCCTGCAAATGCAAACAATG	TCGGAAGCCTCTTTGCAGAT
CHOP	CCACCACACCTGAAAGCAGAA	AGGTGAAAGGCAGGGACTCA
GADD34	TCCCTCATGGGGAGACTGAA	AGCTGTGCGTTCCATTTCCT
Bip	TTCAGCCAATTATCAGCAAACTCT	TTTTCTGATGTATCCTCTTCACCAGT
EDEM	CTACCTGCGAAGAGGCCG	GTTCATGAGCTGCCCACTGA
ATF4	GGGTTCTGTCTTCCACTCCA	AAGCAGCAGAGTCAGGCTTTC
sXBP1	CTGAGTCCGAATCAGGTGCAG	GTCCATGGGAAGATGTTCTGG
GRP94	AAGAATGAAGGAAAAACAGGACAAAA	CAAATGGAGAAGATTCCGCC
PDI	GGATTGCACTGCCAACACAA	AGCTGGTCCTGCTTGTTTCT
Arc	TTGGTAAGTGCCGAGCTGAG	ACGTAGCCGTCCAAGTTGTT
